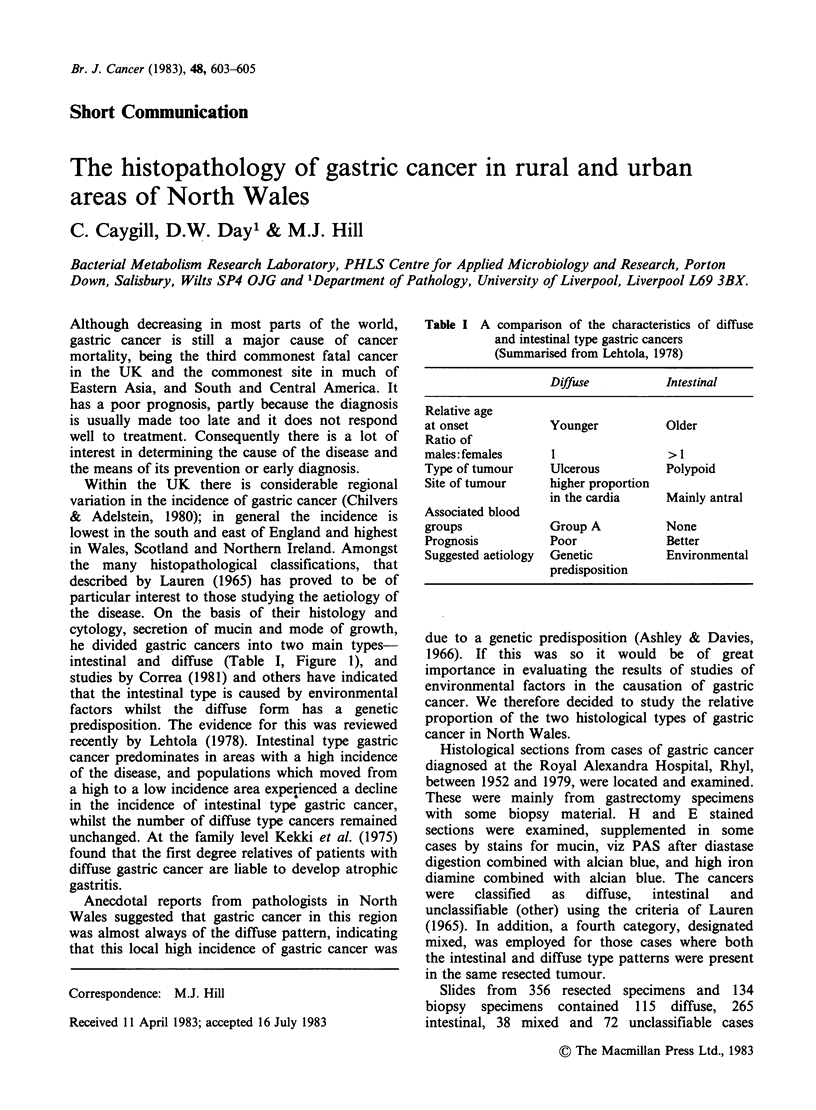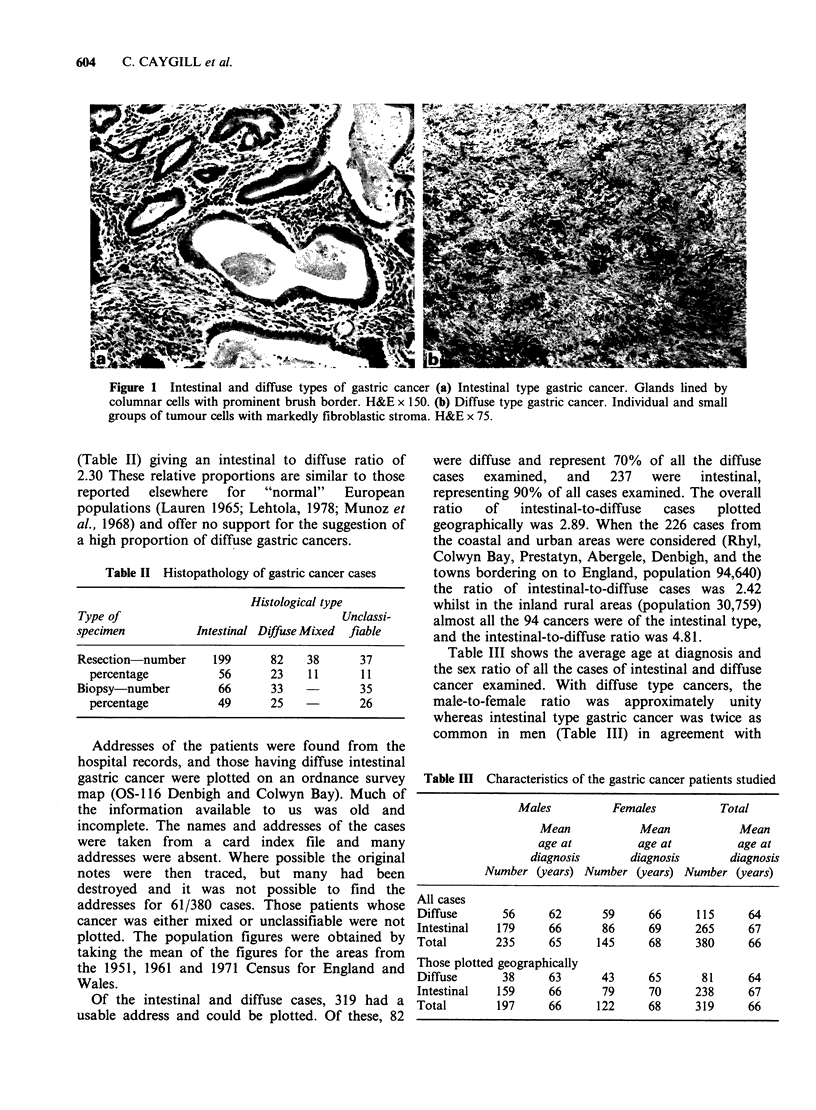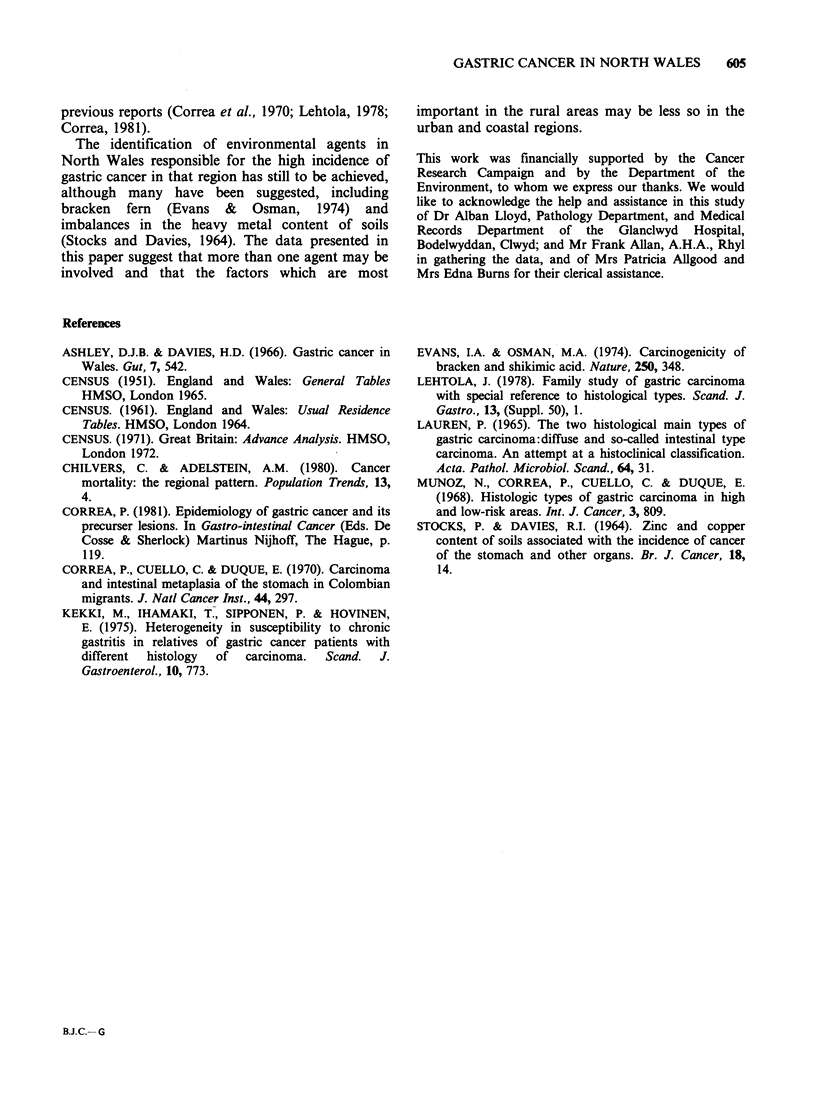# The histopathology of gastric cancer in rural and urban areas of North Wales.

**DOI:** 10.1038/bjc.1983.235

**Published:** 1983-10

**Authors:** C. Caygill, D. W. Day, M. J. Hill

## Abstract

**Images:**


*Br. J. Cancer* (1983), **48**, 603–605

**Short Communication**

### The histopathology of gastric cancer in rural and urban areas of North Wales

C. Caygill, D.W. Day[1] & M.J. Hill

*Bacterial Metabolism Research Laboratory, PHLS Centre for Applied Microbiology and Research, Porton Down, Salisbury, Wilts SP4 OJG and [1]Department of Pathology, University of Liverpool, Liverpool L69 3BX.*

Although decreasing in most parts of the world, gastric cancer is still a major cause of cancer mortality, being the third commonest fatal cancer in the UK and the commonest site in much of Eastern Asia, and South and Central America. It has a poor prognosis, partly because the diagnosis is usually made too late and it does not respond well to treatment. Consequently there is a lot of interest in determining the cause of the disease and the means of its prevention or early diagnosis.

Within the UK there is considerable regional variation in the incidence of gastric cancer (Chilvers & Adelstein, 1980); in general the incidence is lowest in the south and east of England and highest in Wales, Scotland and Northern Ireland. Amongst the many histopathological classifications, that described by Lauren (1965) has proved to be of particular interest to those studying the aetiology of the disease. On the basis of their histology and cytology, secretion of mucin and mode of growth, he divided gastric cancers into two main types—intestinal and diffuse (Table I, Figure 1), and studies by Correa (1981) and others have indicated that the intestinal type is caused by environmental factors whilst the diffuse form has a genetic predisposition. The evidence for this was reviewed recently by Lehtola (1978). Intestinal type gastric cancer predominates in areas with a high incidence of the disease, and populations which moved from a high to a low incidence area experienced a decline in the incidence of intestinal type gastric cancer, whilst the number of diffuse type cancers remained unchanged. At the family level Kekki *et al.* (1975) found that the first degree relatives of patients with diffuse gastric cancer are liable to develop atrophic gastritis.

Anecdotal reports from pathologists in North Wales suggested that gastric cancer in this region was almost always of the diffuse pattern, indicating that this local high incidence of gastric cancer was due to a genetic predisposition (Ashley & Davies, 1966). If this was so it would be of great importance in evaluating the results of studies of environmental factors in the causation of gastric cancer. We therefore decided to study the relative proportion of the two histological types of gastric cancer in North Wales.

**Table I** A comparison of the characteristics of diffuse and intestinal type gastric cancers (Summarised from Lehtola, 1978)

| | *Diffuse* | *Intestinal* |
|---|---|---|
| Relative age at onset | Younger | Older |
| Ratio of males:females | 1 | >1 |
| Type of tumour | Ulcerous | Polypoid |
| Site of tumour | higher proportion in the cardia | Mainly antral |
| Associated blood groups | Group A | None |
| Prognosis | Poor | Better |
| Suggested aetiology | Genetic predisposition | Environmental |

Histological sections from cases of gastric cancer diagnosed at the Royal Alexandra Hospital, Rhyl, between 1952 and 1979, were located and examined. These were mainly from gastrectomy specimens with some biopsy material. H and E stained sections were examined, supplemented in some cases by stains for mucin, viz PAS after diastase digestion combined with alcian blue, and high iron diamine combined with alcian blue. The cancers were classified as diffuse, intestinal and unclassifiable (other) using the criteria of Lauren (1965). In addition, a fourth category, designated mixed, was employed for those cases where both the intestinal and diffuse type patterns were present in the same resected tumour.

Slides from 356 resected specimens and 134 biopsy specimens contained 115 diffuse, 265 intestinal, 38 mixed and 72 unclassifiable cases

Correspondence: M.J. Hill

Received 11 April 1983; accepted 16 July 1983

© The Macmillan Press Ltd., 1983

**Figure 1** Intestinal and diffuse types of gastric cancer **(a)** Intestinal type gastric cancer. Glands lined by columnar cells with prominent brush border. H&E × 150. **(b)** Diffuse type gastric cancer. Individual and small groups of tumour cells with markedly fibroblastic stroma. H&E × 75.

(Table II) giving an intestinal to diffuse ratio of 2.30 These relative proportions are similar to those reported elsewhere for "normal" European populations (Lauren 1965; Lehtola, 1978; Munoz *et al.*, 1968) and offer no support for the suggestion of a high proportion of diffuse gastric cancers.

**Table II** Histopathology of gastric cancer cases

| *Type of specimen* | *Histological type* | | | |
|---|---|---|---|---|
| | *Intestinal* | *Diffuse* | *Mixed* | *Unclassifiable* |
| Resection—number | 199 | 82 | 38 | 37 |
| percentage | 56 | 23 | 11 | 11 |
| Biopsy—number | 66 | 33 | — | 35 |
| percentage | 49 | 25 | — | 26 |

Addresses of the patients were found from the hospital records, and those having diffuse intestinal gastric cancer were plotted on an ordnance survey map (OS-116 Denbigh and Colwyn Bay). Much of the information available to us was old and incomplete. The names and addresses of the cases were taken from a card index file and many addresses were absent. Where possible the original notes were then traced, but many had been destroyed and it was not possible to find the addresses for 61/380 cases. Those patients whose cancer was either mixed or unclassifiable were not plotted. The population figures were obtained by taking the mean of the figures for the areas from the 1951, 1961 and 1971 Census for England and Wales.

Of the intestinal and diffuse cases, 319 had a usable address and could be plotted. Of these, 82 were diffuse and represent 70% of all the diffuse cases examined, and 237 were intestinal, representing 90% of all cases examined. The overall ratio of intestinal-to-diffuse cases plotted geographically was 2.89. When the 226 cases from the coastal and urban areas were considered (Rhyl, Colwyn Bay, Prestatyn, Abergele, Denbigh, and the towns bordering on to England, population 94,640) the ratio of intestinal-to-diffuse cases was 2.42 whilst in the inland rural areas (population 30,759) almost all the 94 cancers were of the intestinal type, and the intestinal-to-diffuse ratio was 4.81.

Table III shows the average age at diagnosis and the sex ratio of all the cases of intestinal and diffuse cancer examined. With diffuse type cancers, the male-to-female ratio was approximately unity whereas intestinal type gastric cancer was twice as common in men (Table III) in agreement with

**Table III** Characteristics of the gastric cancer patients studied

| | *Males* | | *Females* | | *Total* | |
|---|---|---|---|---|---|---|
| | *Number* | *Mean age at diagnosis (years)* | *Number* | *Mean age at diagnosis (years)* | *Number* | *Mean age at diagnosis (years)* |
| All cases | | | | | | |
| Diffuse | 56 | 62 | 59 | 66 | 115 | 64 |
| Intestinal | 179 | 66 | 86 | 69 | 265 | 67 |
| Total | 235 | 65 | 145 | 68 | 380 | 66 |
| Those plotted geographically | | | | | | |
| Diffuse | 38 | 63 | 43 | 65 | 81 | 64 |
| Intestinal | 159 | 66 | 79 | 70 | 238 | 67 |
| Total | 197 | 66 | 122 | 68 | 319 | 66 |

previous reports (Correa *et al.*, 1970; Lehtola, 1978; Correa, 1981).

The identification of environmental agents in North Wales responsible for the high incidence of gastric cancer in that region has still to be achieved, although many have been suggested, including bracken fern (Evans & Osman, 1974) and imbalances in the heavy metal content of soils (Stocks and Davies, 1964). The data presented in this paper suggest that more than one agent may be involved and that the factors which are most important in the rural areas may be less so in the urban and coastal regions.

This work was financially supported by the Cancer Research Campaign and by the Department of the Environment, to whom we express our thanks. We would like to acknowledge the help and assistance in this study of Dr Alban Lloyd, Pathology Department, and Medical Records Department of the Glanclwyd Hospital, Bodelwyddan, Clwyd; and Mr Frank Allan, A.H.A., Rhyl in gathering the data, and of Mrs Patricia Allgood and Mrs Edna Burns for their clerical assistance.

### References

ASHLEY, D.J.B. & DAVIES, H.D. (1966). Gastric cancer in Wales. *Gut*, **7**, 542.

CENSUS (1951). England and Wales: *General Tables* HMSO, London 1965.

CENSUS. (1961). England and Wales: *Usual Residence Tables*. HMSO, London 1964.

CENSUS. (1971). Great Britain: *Advance Analysis*. HMSO, London 1972.

CHILVERS, C. & ADELSTEIN, A.M. (1980). Cancer mortality: the regional pattern. *Population Trends*, **13**, 4.

CORREA, P. (1981). Epidemiology of gastric cancer and its precurser lesions. In *Gastro-intestinal Cancer* (Eds. De Cosse & Sherlock) Martinus Nijhoff, The Hague, p. 119.

CORREA, P., CUELLO, C. & DUQUE, E. (1970). Carcinoma and intestinal metaplasia of the stomach in Colombian migrants. *J. Natl Cancer Inst.*, **44**, 297.

KEKKI, M., IHAMAKI, T., SIPPONEN, P. & HOVINEN, E. (1975). Heterogeneity in susceptibility to chronic gastritis in relatives of gastric cancer patients with different histology of carcinoma. *Scand. J. Gastroenterol.*, **10**, 773.

EVANS, I.A. & OSMAN, M.A. (1974). Carcinogenicity of bracken and shikimic acid. *Nature*, **250**, 348.

LEHTOLA, J. (1978). Family study of gastric carcinoma with special reference to histological types. *Scand. J. Gastro.*, **13**, (Suppl. 50), 1.

LAUREN, P. (1965). The two histological main types of gastric carcinoma: diffuse and so-called intestinal type carcinoma. An attempt at a histoclinical classification. *Acta. Pathol. Microbiol. Scand.*, **64**, 31.

MUNOZ, N., CORREA, P., CUELLO, C. & DUQUE, E. (1968). Histologic types of gastric carcinoma in high and low-risk areas. *Int. J. Cancer*, **3**, 809.

STOCKS, P. & DAVIES, R.I. (1964). Zinc and copper content of soils associated with the incidence of cancer of the stomach and other organs. *Br. J. Cancer*, **18**, 14.